# Cancer of the endometrium

**DOI:** 10.1054/bjoc.2000.1760

**Published:** 2001-05

**Authors:** A Brémond, A Bataillard, L Thomas, J L Achard, B Fervers, E Fondrinier, J Lansac, C Bailly, S Hoffstetter, J P Basuyau, J d'Anjou, P Descamps, F Farsi, J P Guastalla, F Laffargue, J F Rodier, P Vincent, J Pigneux

**Affiliations:** Centre Léon Bérard, Lyon; Cancer Research Campaign, Paris; Institut Bergonié, Bordeaux; Centre Jean Perrin, Clermont-Ferrand; Centre Paul Papin, Angers; CHU Bretonneau, Tours; Centre Alexis Vautrin, Nancy; Centre Henri Becquerel, Rouen; CHU, Angers; CHU, Hôpital Arnaud de Villeneuve, Montpellier; Centre Paul Strauss, Strasbourg; Clinique Sainte-Catherine, Avignon, France


					In 1995 the number of endometrial cancers diagnosed was esti-
mated to be 4600, corresponding to a standardized incidence rate
of 13.6 cases per 100 000 women in Europe. In the same year
endometrial cancer was responsible for approximately 1200 deaths
in France and it has become the third most common cancer in
women after breast and colorectal tumours.
This document concerns the management of cancer of the
uterine body but does not include sarcomas of the uterus.
These guidelines were validated in January 2000 and an update
is planned in 2003.
DIAGNOSIS
Hysteroscopy and transvaginal ultrasonography are the best initial
examinations. The findings must be confirmed by biopsy (stan-
dard). Outpatient biopsy using Pipelle endometrial sampling is
only useful if positive. It improves the specificity of a transvaginal
ultrasound (standard). Fractional curettage gives the diagnosis
in 95% of cases (standard). CA125 is of no diagnostic value
(standard).
STAGING
Although the primary treatment of endometrial carcinoma is
usually surgery, preoperative imaging has an important role in the
evaluation of operability (Figure 1):
 in rare cases where there is a contra-indication to surgery
 to detect locally advanced stages not suspected on clinical
examination (for example in obese patients or in those difficult
to examine)
 to assess iliac and/or para-aortic lymph nodes
 to assess pre-surgical prognostic factors (for example exten-
sion to the myometrium and to the cervix and thus the risk of
nodal involvement).
Staging investigations must be planned by a multidisciplinary
team (standard) and be adapted to the treatment strategy used by
the team (standard). To evaluate local regional extension, a preop-
erative ultrasound, (which is not standard), is usually done
(option). CT scanning is the best way to detect para-aortic nodal
involvement (option). Literature review shows a small advantage
for MRI over transvaginal ultrasonography for staging penetration
of the myometrium. Ultrasonography is more readily available and
less costly however (option). No examination is sufficiently sensi-
tive and specific to distinguish between stage I and stage II
disease. CA125 may predict for extra-uterine extension at levels
>35 U ml-1
.
CLASSIFICATION
The pathological report must specify the precise origin of the
tumour, its size and macroscopic features. It must document the
extent of macroscopic infiltration of the myometrium, and if there
is invasion of the cervix, parametrium and/or adnexae (standard).
The diagnosis of endometrial carcinoma is based on histological
examination. This determines both the histological type and
grading of the tumour (standard). Immunohistochemistry and
evaluation of hormone receptors may be carried out (option). In
the case of diagnostic difficulty, immunohistochemistry is recom-
mended. Use of the FIGO 1988 classification is standard (stan-
dard).
PROGNOSTIC FACTORS
The following prognostic factors must be determined: FIGO
classification, grade, differentiation, cervical invasion, depth of
myometrial invasion, involvement of pelvic nodes, ovarian
involvement, peritoneal cytology and histological type (papillary
serous and clear cell carcinomas tend to behave more aggres-
sively) (standard). A number of other prognostic factors may be
considered: involvement of para-aortic nodes, initial CA125,
hormone receptors, ploidy, growth factors (option). The use of
FACS (fluorescent activation cytology scanning) is not currently
recommended outside clinical research protocols. CA125 is corre-
lated with prognosis but it is impossible to know if the assay
provides any real benefit to the patient. It is of little value after
treatment. The value of other markers is not yet clear.
SURGICAL TREATMENT OF STAGE I AND II
DISEASE
Surgery must always include the exploration, systematic inspec-
tion and palpation of the entire abdomen (standard) (Figure 2). All
abnormal areas must be biopsied (standard). A sample must be
taken for peritoneal cytology (standard, expert agreement). The
hysterectomy must be at least total and extrafascial (standard, level
of evidence B) with bilateral salpingo-oophorectomy (BSO) (stan-
dard, level of evidence B) (Figure 3). A modified radical hysterec-
tomy (Piver type II) is undertaken for stage II cancers with
macroscopic cervical lesions (standard) (Figure 4).
Lymphadenectomy can be undertaken by laparotomy or
laparoscopy (option, level of evidence B). Hysterectomy can be by
laparotomy, by the vaginal route or by laparoscopy (option, level
of evidence B). An omentectomy is undertaken in the case of
serous papillary forms (level of evidence B). When preoperative
Cancer of the endometrium
A Bremond1
, A Bataillard1,2
, L Thomas3
, JL Achard4
, B Fervers1,2
, E Fondrinier5
, J Lansac6
, C Bailly4
, S Hoffstetter7
,
JP Basuyau8
, J d'Anjou8
, P Descamps9
, F Farsi1
, JP Guastalla1
, F Laffargue10
, JF Rodier11
, P Vincent12
and J Pigneux3
1
Centre Leon Berard, Lyon; 2
FNCLCC, Paris; 3
Institut Bergonie, Bordeaux; 4
Centre Jean Perrin, Clermont-Ferrand; 5
Centre Paul Papin, Angers; 6
CHU
Bretonneau, Tours; 7
Centre Alexis Vautrin, Nancy; 8
Centre Henri Becquerel, Rouen; 9
CHU, Angers; 10
CHU, Hopital Arnaud de Villeneuve, Montpellier; 11
Centre
Paul Strauss, Strasbourg; 12
Clinique Sainte-Catherine, Avignon, France
31
British Journal of Cancer (2001) 84(Supplement 2), 31-36
(c) 2001 FNCLCC
doi: 10.1054/ bjoc.2001.1760, available online at http://www.idealibrary.com on
staging has failed to show macroscopic invasion of the cervix, a
modified radical hysterectomy (Piver type II) gives no added
benefit over a simple hysterectomy.
PLACE OF PELVIC LYMPHADENECTOMY IN
STAGE I AND II DISEASE
Pelvic lymphadenectomy can be done by laparotomy or by
laparoscopy (options). Pelvic lymphadenectomy (option) should
not be undertaken when there are bad prognostic factors (i.e. grade
3 pathology, greater than 50% infiltration of myometrium, stage II
disease) that will necessitate postoperative radiotherapy (Figure 4).
Pelvic lymphadenectomy is undertaken if the patient is of good
performance status and if surgery is likely to be uncomplicated
(Figures 3 and 4). Lymphadenectomy must not be undertaken in a
patient of poor performance status; the uncertainty as to any
benefit on survival does not justify the operative risk. Pelvic
lymphedenectomy is recommended by the International
Federation of Obstetricians and Gynaecologists (FIGO) for the
purposes of precise staging. Published data do not differentiate
between the benefit of routine extended pelvic lymphadenectomy
done in order to obtain optimum histopathological information,
and a more selective approach whereby lymphadenectomy is only
done in patients with a good prognosis. Randomized studies with
comparable treatment in each sub-group are necessary to clarify
this.
PLACE OF PARA-AORTIC LYMPHADENECTOMY
IN STAGE I AND II DISEASE
Para-aortic lymphadenectomy does not constitute standard therapy
in cancer of the endometrium. Excision of enlarged nodes can be
32 A Bremond et al
British Journal of Cancer (2001) 84 (Supplement 2), 31-36 (c) 2001 FNCLCC
Evaluation of operability
Standard
evaluation of operability based on:
* tumour criteria
* patient criteria
Operable disease?
Standard
surgery, radical or adapted to the tumour extent
Options
when cervical curettage is positive (proven stage II):
preoperative radiotherapy + brachytherapy or
brachytherapy
Is radical surgery possible?
yes
yes
no
no
Standard
postoperative irradiation
Options
* post-operative external beam
irradiation
* post-operative brachytherapy
* post-operative brachytherapy and
external beam irradiation
Operable disease,
radical surgery
possible
see Figure 2
Follow-up
Surgery impossible
see Figure 8
Figure 1 Evaluation of operability for cancer of the endometrium
an option. Para-aortic lymphadenectomy by laparotomy or
laparoscopy (for those teams trained in this technique) can be
undertaken (option) (Figure 4). Selective lymphadenectomy of
enlarged para-aortic nodes is recommended. Routine para-aortic
nodal clearance is not recommended. Although the recommenda-
tions of the Gynaecological Oncology Group (GOG) argue in
favour of routine para-aortic lymphadenectomy, primarily for the
purpose of staging, the following factors need to be taken into
consideration:
 extent of the surgery
 presence of altered patient anatomy (due to age, or multiple
concomitant disorders)
 rarity of isolated para-aortic invasion
 high predictive value of the involvement of pelvic nodes by
para-aortic involvement
 correlation between nodal involvement and other main prog-
nostic factors (invasion of the myometrium, ovaries, occult
peritoneal disease, etc)
 controversy over the efficacy of adjuvant therapies (with the
possible exception of occult nodal invasion).
The importance of these factors awaits confirmation from other
studies. Experimental studies using injected coloured markers are
underway to determine the pathways of uterine lymphatic drainage
in order to facilitate selective lymphadenectomy.
SURGERY FOR STAGES III AND IV
Clinical stage III and IV cancers of the endometrium carry a bad
prognosis. If the performance status of the patient permits it,
cytoreductive surgery remains the best way to improve overall
survival. Radical surgery must be the intention (standard) (Figures 1
and 5). Resection, as extensive as possible, followed by radiotherapy,
or sub-optimal surgery followed by irradiation are possibilities
Cancer of the endometrium 33
British Journal of Cancer (2001) 84 (Supplement 2), 31-36
(c) 2001 FNCLCC
Operable disease, radical surgery possible
Standards
* laparotomy
* full surgical staging
Surgical staging
Standards
* inspection and palpation of the peritoneum, nodes and the omentum
* biopsy/excision of abnormal areas
* cytology
Confirmed stage I
disease
see Figure 3
Probable stage I or II or
definite stage II
see Figure 4
Stage III
see Figure 5
Stage IV
see Figure 7
Figure 2 Management of operable disease: surgical staging
Standard
hysterectomy with bilateral oophorectomy
Option
pelvic lymphadenectomy
Postoperative staging according to FIGO,
determination of histoprognostic factors
ADDITIONAL TREATMENT
STAGE IA
Standard
follow-up
Option
vaginal brachytherapy for grades 3 tumours, or extended
to the whole uterine cavity or disease localized
near the cervix
STAGE IB grade 1 or 2
Standard
there is no standard
Options
* vaginal brachytherapy
* follow-up
STAGE IB grade 3, STAGE IC
Standard
there is no standard
Options
* pelvic radiotherapy  brachytherapy boost
* vaginal brachytherapy
Follow-up
Confirmed stage I disease
Figure 3 Treatment of stage I disease confirmed by surgical staging
Probable Stage I or II disease,
Definite stage II
Standards
* modified radical hysterectomy (Piver 2)
* pelvic clearance
Option
* para-aoritc lymphadenectomy
* stage II (proven):
- pelvic radiotherapy + uterovaginal brachytherapy } + total hysterectomy
- brachytherapy } with bilateral oophorectomy
Postoperative staging according to FIGO
Determination of histoprognostic factors
ADJUVANT TREATMENT
(if preoperative radiotherapy has not been given)
STAGE IIA
Standards
* myometrial invasion <50% of grade 1 or 2: vaginal
brachytherapy
* myometrial invasion> 50% of grade 3: pelvic radiotherapy
and brachytherapy boost
STAGE IIB
Standard
pelvic radiotherapy and brachytherapy boost
Follow-up
Figure 4 Treatment of stage I or II disease and confirmed stage II disease
(options) (Figures 1, 5 and 6). It is recommended that cytoreduc-
tion surgery be carried out as follows: paramedial approach, total
hysterectomy with BSO, colpectomy extending to adjacent
healthy tissue, excision of pelvic and para-aortic nodes that are
macroscopically involved, excision of free omentum in the case of
ovarian involvement and location by clips of unresectable macro-
scopically involved nodes (Figures 5 and 6).
If the performance status of the patient is poor, a total hysterec-
tomy plus BSO by an abdominal approach is preferable to treat-
ment with radiotherapy alone.
SURGICAL TREATMENT OF STAGE IV DISEASE
Cytoreduction surgery is undertaken with a paramedial approach,
total hysterectomy plus BSO, gut resection (if this allows for
complete resection or if it is necessary to avoid obstruction) and
partial or total bladder resection with urinary diversion (standard)
(Figure 7).
RADIOTHERAPY
Additional treatment for stage I disease after surgery
For grade 1 and 2 stage IA tumours, follow-up alone is standard
(Figure 3). Vaginal brachytherapy (option) can be undertaken for
grade 3 stage IA disease, or for tumours localized adjacent to the
cervix or involving the whole uterine cavity (Figure 3). For grade
1 and 2 stage IB tumours, the options are vaginal brachytherapy or
follow-up alone (Figure 3). For grade 3, stage IB tumours and
stage IC disease whatever the grade is or may be, there are two
treatment options (Figure 3): external pelvic radiotherapy with or
without a vaginal brachytherapy boost (level of evidence B) or
vaginal brachytherapy (level of evidence C). Preoperative radio-
therapy (either external or brachytherapy) is not recommended for
stage I disease, as it cannot be planned according to specific histo-
prognostic factors of the tumour or to its exact extent and would
therefore constitute overtreatment for some stage I tumours.
Additional treatment for stage II disease
When stage II disease has been proven by positive endocervix or
cervix biopsy, there are two options; external radiotherapy with
brachytherapy or brachytherapy followed by surgery, or surgery,
34 A Bremond et al
British Journal of Cancer (2001) 84 (Supplement 2), 31-36 (c) 2001 FNCLCC
Stage III
Radical surgery possible?
SURGERY
Standard
total hysterectomy with oophorectomy and pelvic lymphadenectomy
Options
* omentectomy if ovaries involved
* para-aortic nodal clearance
ADDITIONAL TREATMENT
STAGE IIIA
Standard
there is no standard
Options
* postoperative pelvic radiotherapy
* abdomino-pelvic radiotherapy
* therapeutic trial of chemotherapy
STAGE IIIB
Standard
pelvic external beam irradiation with brachytherapy, if possible
STAGE IIIC, pelvic nodes involved
Standard
post-operative pelvic radiotherapy brachytherapy boost
Options
* abdomino-pelvic radiotherapy
* extended postoperative radiotherapy (pelvic and para-aortic)
STAGE IIIC, para-aortic nodes involved
Standard
extended postoperative radiotherapy (pelvic and para-aortic)  brachytherapy
Follow-up
Stage III when radical
surgery is not possible
see Figure 6
yes no
Figure 5 Treatment of stage III disease
Stage III disease where radical surgery is impossible
SURGERY
Standard
debulking surgery:
* total hysterectomy with salpingo-oophorectomy
* bowel resection if possible
* partial or total bladder resection if possible
Options
* total hysterectomy + cervix ablation = radical
hysterectomy
* para-aortic nodal clearence
ADDITIONAL TREATMENT
Standard
there is no standard
Options
* postoperative external radiotherapy  brachytherapy
* therapeutic trial of hormone therapy or chemotherapy
Follow-up
Figure 6 Treatment of stage III disease where radical surgery is impossible
Standard
anterior or posterior pelvectomy depending on location with pelvic clearance
Options
* post-operative pelvic radiotherapy  brachytherapy
* para-aortic nodal clearance
* clinical trial of hormone therapy or chemotherapy
Stage IV
Stage IVa Stage IVb
Peritoneal disease?
Management of metastatic
disease
Follow-up
no yes
Figure 7 Treatment of stage IV disease
as primary treatment, followed by adjuvant radiotherapy given
according to prognostic factors (Figures 1 and 4). If involvement
of the cervix is not confirmed, primary surgery is recommended.
Postoperative vaginal brachytherapy is given for stage IIA
tumours if the penetration of the myometrium is less than 50% or
if the tumour is grade 1 or 2 (standard) (Figure 4). When the
myometrial penetration is more than 50% or for grade 3 disease,
external radiotherapy with a brachytherapy boost has to be under-
taken routinely (standard) (Figure 4). After primary surgery for
stage IIB disease, postoperative external pelvic radiotherapy with
a brachytherapy boost must routinely be undertaken (standard)
(Figure 4).
Additional treatment for stage III disease
For stage IIIA disease involving the ovaries only or with positive
peritoneal cytology only, either external pelvic radiotherapy or
abdomino-pelvic radiotherapy is advisable (option) (Figure 5). For
stage IIIA tumours involving several extrauterine sites, standard
treatment is abdomino-pelvic radiotherapy (standard), with addi-
tional medical treatment in certain patients (option). For stage IIIB
tumours, postoperative external radiotherapy with brachytherapy
(if possible) should be undertaken (standard) (Figure 5). For stage
IIIC tumours, (involvement of pelvic nodes), standard treatment is
external pelvic radiotherapy followed by a brachytherapy boost
(standard) (Figure 5). Extended-field radiotherapy to para-aortic
nodes is an option. If there are extra-uterine sites involved
abdomino-pelvic radiotherapy is recommended (option). For stage
IIIC tumours involving para-aortic nodes, extended external radio-
therapy including pelvic and para-aortic nodes with or without
brachytherapy is recommended (Figure 5).
Treatment of inoperable disease
Standard treatment for inoperable stage I and II disease is external
radiotherapy and brachytherapy (standard) (Figure 8). There are
three possible options, radiotherapy alone, brachytherapy alone
for stage I, or radiotherapy plus medical treatment (options). For
patients with inoperable stage III or IV disease, treatment is often
symptomatic, combining palliative external radiotherapy with
medical treatment (Figure 8).
Complications of radiotherapy
Late complications of external radiotherapy are most commonly
gastrointestinal. Their frequency is correlated with the dose deliv-
ered to critical organs. The complications of postoperative
brachytherapy include rectal injury and vaginal stenosis. These
complications are closely linked to the dose delivered and to the
length of vagina irradiated. If radiotherapy is the only treatment,
both external radiotherapy and brachytherapy must be carefully
planned to give good local control with the minimum of complica-
tions.
ADJUVANT HORMONE THERAPY
There is no evidence to support the use of adjuvant hormone
therapy (level of evidence A) Adjuvant hormonal therapy (option)
should only be given within the context of a therapeutic trial (stan-
dard) (Figures 6, 7 and 8).
CHEMOTHERAPY IN CANCER OF THE
ENDOMETRIUM
Chemotherapy can be used for palliation (option). Doxorubicin
and cisplatin are the drugs most commonly used. New Drugs are in
the process of evaluation (option). The role of chemotherapy in
metastatic cancer of the endometrium is limited in that the rate of
response is less than 50%. Any benefit with respect to survival or
quality of life has yet to be determined. No randomized study has
compared chemotherapy with best supportive care.
Adjuvant chemotherapy
No study has shown a benefit from adjuvant chemotherapy in
cancer of the endometrium. Few randomized trials have been
published; some have included only a small number of patients,
others have not given final results. The inclusion criteria for trial
entry are variable, making it difficult to compare results. There are
prospective uncontrolled studies, but the small number of patients
and the absence of randomization prevents any definitive conclu-
sions to be made as to the efficacy of adjuvant chemotherapy.
Adjuvant chemotherapy should be given within therapeutic trials
(option) (Figures 6, 7 and 8).
FOLLOW-UP
In the absence of specific symptoms or signs, follow-up is based
on general and gynaecological examination (standard). The ideal
timing of follow-up examinations has not been formally estab-
lished; once every 6 months for the first 3 years then yearly is
sufficient (standard). All patients presenting with symptoms
Cancer of the endometrium 35
British Journal of Cancer (2001) 84 (Supplement 2), 31-36
(c) 2001 FNCLCC
Surgery impossible
Stage?
no yes
Stages I and II Stages III and IV
Standard
external radiotherapy then brachytherapy, if possible
Options
* brachytherapy alone for stage I
* external radiotherapy alone
Standard
there is no standard
Options
* external radiotherapy and brachytherapy
* external radiotherapy
* external radiotherapy and hormone therapy
* therapeutic trial of hormone therapy and chemotherapy
Follow-up
Figure 8 Treatment of inoperable disease
should have a full work-up (standard). There is no indication to
carry out supplementary examinations looking for relapse or
metastases in the absence of clinical signs. The CA125 assay is
one examination which enables early diagnosis of recurrence, but
this has no proven benefit with respect to prognosis and cannot be
recommended as routine. It has not been demonstrated that
hormonal replacement therapy in women of low risk with difficult
menopausal symptoms increases the risk of recurrence or of
metastases (option). Prospective studies are necessary before
hormone replacement therapy can be recommended for women
previously treated for carcinoma of the endometrium.
INTERNAL REVIEWERS
B Asselain (Institut Curie, Paris), I Barillot (Centre Georges-
Francois Leclerc, Dijon), V Beckendorf (Centre Alexis Vautrin,
Nancy), JM Bidart (Institut Gustave Roussy, Villejuif), N Callet
(Centre Rene Huguenin, Saint-Cloud), D Castaigne (Institut
Gustave Roussy, Villejuif), B Castelain (Centre Oscar Lambret,
Lille), C Charra-Brunaud (Centre Alexis Vautrin, Nancy),
A Chevalier (Centre Oscar Lambret, Lille), C Cohen-Solal (Centre
Rene Huguenin, Saint-Cloud), H Crouet (Centre Francois
Baclesse, Caen), H Cure (Centre Jean Perrin, Clermont-Ferrand),
N Daly-Schveitzer (Institut Claudius Regaud, Toulouse), A De la
Rochefordiere (Institut Curie, Paris), P Dessogne (Centre Henri
Becquerel, Rouen), D Di Stefano-Louineau (Institue Paoli
Calmettes, Marseille), JB Dubois (Centre Val d'Aurelle,
Montpellier), F Eisinger (Institut Paoli Calmettes, Marseille),
F. Eschwege (Institut Gustave Roussy, Villejuif), M Fabbro
(Centre Val d'Aurelle, Montpellier), A Floquet (Institut Bergonie,
Bordeaux), A Gerbaulet (Institut Gustave Roussy, Villejuif),
L Gladieff (Centre Claudius Regaud, Toulouse), L Gonzague-
Casabianca (Institut Paoli Calmettes, Marseille), A Goupil (Centre
Rene Huguenin, Saint-Cloud), C Haie-Meder (Institut Gustave
Roussy, Villejuif), JF Heron (Centre Francois Baclesse,
Caen), JC Horiot (Centre Georges-Francois Leclerc, Dijon),
G Houvenaeghel (Institut Paoli Calmettes, Marseille),
J Jacquemier (Institut Paoli Calmettes, Marseille), F Joly (Centre
Francois Baclesse, Caen), P Kerbrat (Centre Eugene Marquis,
Rennes), E Lartigau (Institut Gustave Roussy, Villejuif), S Lasry
(Centre Rene Huguenin, Saint-Cloud), C Lasset (Centre Regional
Leon Berard, Lyon), E Leblanc (Centre Oscar Lambret, Lille),
A Lebrun-Jezekova (Institut Jean Godinot, Reims), J Leclere
(Institut Gustave Roussy, Villejuif), V Le Doussal (Centre Rene
Huguenin, Saint-Cloud), C Lhomme (Institut Gustave Roussy,
Villejuif), P Martel (Centre Claudius Regaud, Toulouse), F Mayer
(Centre Georges-Francois Leclerc, Dijon), M Namer (Centre
Antoine Lacassagne, Nice), TD Nguyen (Institut Jean Godinot,
Reims), P Pautier (Institut Gustave Roussy, Villejuif), T Petit
(Centre Paul Strauss, Strasbourg), MF Pichon (Centre Rene
Huguenin, Saint-Cloud), J Pigneux (Institut Bergonie, Bordeaux),
N Quetin (Centre Paul Strauss, Strasbourg), M Resbeut (Institut
Paoli Calmettes, Marseille), M Rios (Centre Alexis Vautrin,
Nancy), H Sancho-Garnier (Centre Epidaure, Centre Val
d'Aurelle, Montpellier), X Sastre-Garau (Institut Curie, Paris),
E Stockle (Institut Bergonie, Bordeaux), E Teissier (Centre
Antoine Lacassagne, Nice), F Ternier (Institut Paoli Calmettes,
Marseille), L Thomas (Institut Bergonie, Bordeaux), P Troufleeau
(Centre Alexis Vautrin, Nancy), P Vennin (Centre Oscar Lambret,
Lille), P Viens (Institut Paoli Calmettes, Marseille), B Weber
(Centre Alexis Vautrin, Nancy) and P Zlatoff (Centre regional
Leon Berard, Lyon).
EXTERNAL REVIEWERS
L Aimard (Residence du Parc, Marseille), P Azuar (CHG-Hopital
Grasse, Grasse), C Bergeron (Laboratoire Pasteur Cerba, Cergy
Pontoise), B Blanc (Societe Francaise de Colposcopie, Hopital de la
Conception, Marseille), M Bolla (CHU-Hopital Nord, Grenoble), J
Bonneterre (Societe Francaise du Cancer, Paris), G Brun (Pessac), G
Calais (CHU Bretonneau, Tours), R Chomier (Clinique Sainte-
Therese, Bron), J-P Cleophax (Centre Hospitalier, Montmorency), D
Collin (Centre Hospitalier, Hagueneau), M Conte (Clinique
Beauregard, Marseille), G Crepin (Hopital Jeanne de Flandre, Lille),
G de Laroche (Clinique Mutualiste de la Digonniere, Saint-Etienne),
JP Dujols (Centre de Radiotherapie et d'Oncologie Medicale, Pau), L
Frappart (CHU, Lyon), JP Gerard (Centre hospitalier Lyon Sud,
Pierre Benite), J-Y Grall (Hopital Sud, Rennes), M Istier (Cabinet
Medical, Lyon), Y Kessler (Societe Francaise de Cancerologie
Privee, Maxeville), H Lauche (Clinique Clementville, Montpellier),
JP Le Bourgeois (Hopital Henri Mondor, Creteil), O Le Floch
(Hopital Bretonneau, Tours), JP Lefranc (Hopital de la Pitie
Salpetriere, Paris), B Maria (College National des Gynecologues et
Obstetriciens Francais, Paris), P. Martin (Societe Francaise de
Radiotherapie Oncologique, Lille), JJ Mazeron (SFRO, Centre
Antoine Beclere, Paris), B. May (Nancy), JL Misset (Hopital Paul
Brousse, Villejuif), J Mouchel (Rue d'Alger, Le Mans), B Paniel
(Centre Hospitalier Intercommunal, Creteil), RM Parache (Societe
Francaise de Gynecopathologie, Vandoeuvre-Les-Nancy), P Piedbois
(Hopital Henri Mondor, Creteil), D Querleu (CHU-Pavillon Paul
Gelle, Roubaix), JM Reme (Hopital de la Grave, Toulouse), P
Revelin (Centre Hospitalier, Roanne), P Romestaing (Centre
Hospitalier Lyon Sud, Pierre-Benite), J Salvat (Centre Hospitalier,
Thonon les Bains), M Unterreiner (Clinique Claude Bernard, Metz),
S Verbaere (Cabinet medical, Saint-Etienne) and E Voog (Hopital
Edouard Herriot, Lyon).
36 A Bremond et al
British Journal of Cancer (2001) 84 (Supplement 2), 31-36 (c) 2001 FNCLCC

				

